# Comparison of Two-Dimensional- and Three-Dimensional-Based U-Net Architectures for Brain Tissue Classification in One-Dimensional Brain CT

**DOI:** 10.3389/fncom.2021.785244

**Published:** 2022-01-10

**Authors:** Meera Srikrishna, Rolf A. Heckemann, Joana B. Pereira, Giovanni Volpe, Anna Zettergren, Silke Kern, Eric Westman, Ingmar Skoog, Michael Schöll

**Affiliations:** ^1^Wallenberg Centre for Molecular and Translational Medicine, University of Gothenburg, Gothenburg, Sweden; ^2^Department of Psychiatry and Neurochemistry, Institute of Physiology and Neuroscience, University of Gothenburg, Gothenburg, Sweden; ^3^Department of Medical Radiation Sciences, Institute of Clinical Sciences, Sahlgrenska Academy, Gothenburg, Sweden; ^4^Division of Clinical Geriatrics, Department of Neurobiology, Care Sciences and Society, Karolinska Institutet, Stockholm, Sweden; ^5^Memory Research Unit, Department of Clinical Sciences, Malmö Lund University, Mälmo, Sweden; ^6^Department of Physics, University of Gothenburg, Gothenburg, Sweden; ^7^Neuropsychiatric Epidemiology, Institute of Neuroscience and Physiology, Sahlgrenska Academy, Centre for Ageing and Health (AgeCap), University of Gothenburg, Gothenburg, Sweden; ^8^Region Västra Götaland, Sahlgrenska University Hospital, Psychiatry, Cognition and Old Age Psychiatry Clinic, Gothenburg, Sweden; ^9^Dementia Research Centre, Institute of Neurology, University College London, London, United Kingdom; ^10^Department of Clinical Physiology, Sahlgrenska University Hospital, Gothenburg, Sweden

**Keywords:** brain image segmentation, CT, MRI, deep learning, convolutional neural networks

## Abstract

Brain tissue segmentation plays a crucial role in feature extraction, volumetric quantification, and morphometric analysis of brain scans. For the assessment of brain structure and integrity, CT is a non-invasive, cheaper, faster, and more widely available modality than MRI. However, the clinical application of CT is mostly limited to the visual assessment of brain integrity and exclusion of copathologies. We have previously developed two-dimensional (2D) deep learning-based segmentation networks that successfully classified brain tissue in head CT. Recently, deep learning-based MRI segmentation models successfully use patch-based three-dimensional (3D) segmentation networks. In this study, we aimed to develop patch-based 3D segmentation networks for CT brain tissue classification. Furthermore, we aimed to compare the performance of 2D- and 3D-based segmentation networks to perform brain tissue classification in anisotropic CT scans. For this purpose, we developed 2D and 3D U-Net-based deep learning models that were trained and validated on MR-derived segmentations from scans of 744 participants of the Gothenburg H70 Cohort with both CT and T1-weighted MRI scans acquired timely close to each other. Segmentation performance of both 2D and 3D models was evaluated on 234 unseen datasets using measures of distance, spatial similarity, and tissue volume. Single-task slice-wise processed 2D U-Nets performed better than multitask patch-based 3D U-Nets in CT brain tissue classification. These findings provide support to the use of 2D U-Nets to segment brain tissue in one-dimensional (1D) CT. This could increase the application of CT to detect brain abnormalities in clinical settings.

## Introduction

X-ray CT and MRI are the most frequently used modalities for structural assessment in neurodegenerative disorders (Wattjes et al., 2009; Pasi et al., 2011). MRI scans are commonly used for image-based tissue classification to quantify and extract atrophy-related measures from structural neuroimaging modalities (Despotović et al., 2015). Many software tools exist to perform automated brain segmentation in MR images, mainly for research purposes (Zhang et al., 2001; Ashburner and Friston, 2005; Cardoso et al., 2011; Fischl, 2012). Currently, CT scanning is used for the visual assessment of brain integrity and the exclusion of copathologies in neurodegenerative diseases (Musicco et al., 2004; Rayment et al., 2016). However, several studies suggest that visual assessment of brain volume changes derived from CT could also be used as the predictors of dementia, displaying comparable diagnostic properties to visual ratings of MRI scans (Sacuiu et al., 2018; Thiagarajan et al., 2018). In comparison with MR imaging, CT scanning is faster, cheaper, and more widely available. Despite these advantages, automated tissue classification in head CT is largely underexplored.

Brain tissue segmentation on CT is a challenging task due to lower soft-tissue contrast compared to MRI. Many existing CT data and some scanners collect anisotropic CT data or one-dimensional (1D) CT images. Several studies have recommended the quantification of brain tissue classes in CT using MR segmentation methods (Manniesing et al., 2017; Cauley et al., 2018), general image thresholding (Gupta et al., 2010) or segmentation methods (Aguilar et al., 2015), or probabilistic classification using Hounsfield units (HU) (Kemmling et al., 2012).

More recent studies have also started exploring the usage of deep learning in brain image segmentation (Akkus et al., 2017; Chen et al., 2018; Henschel et al., 2020; Zhang et al., 2021) mainly using MR images. For this purpose, fully convolutional neural networks (CNNs), residual networks, U-Nets, and recurrent neural networks are commonly used architectures. U-Net, developed in 2015, is a CNN-based architecture for biomedical image segmentation that performs segmentation by classifying at every pixel/voxel (Ronneberger et al., 2015). Many studies have used U-Nets to perform semantic segmentation in MRI (Wang et al., 2019; Wu et al., 2019; Brusini et al., 2020; Henschel et al., 2020; Zhang et al., 2021) and few in CT (Van De Leemput et al., 2019; Akkus et al., 2020). An influential and challenging aspect of deep learning-based studies, especially segmentation-based tasks is the selection of data and labels used for training (Willemink et al., 2020).

The two-dimensional (2D)-based deep learning models use 2D functions to train and predict segmentation maps for a single slice. To predict segmentation maps for full volumes, 2D models take predictions one slice at a time. The 2D model functions can capture context across the height and width of the slice to train and make predictions. The three-dimensional (3D)-based deep learning architectures can capture interslice context. However, this comes at a computational cost due to the increased number of parameters used by the models. To accommodate computational needs, patches are processed instead of whole 3D volumes (Alom et al., 2019). The 3D patch-based processing is a useful method for processing large 3D volumes in 3D image classification and segmentation using deep learning, especially in MR image processing for deep learning (Baid et al., 2018; Largent et al., 2019).

Previously, we conducted a study exploring the possibility of using MR-derived brain tissue class labels to train deep learning models to perform brain tissue classification in head CTs (Srikrishna et al., 2021). We showed that 2D U-Nets could be successfully trained to perform automated segmentation of gray matter (GM), white matter (WM), cerebrospinal fluid (CSF), and intracranial volume in head CT. In this study, we planned to explore if incorporating interslice information using 3D deep learning models could improve CT brain segmentation performance. Patch-based 3D segmentation networks have been successfully used to develop 3D deep learning-based MRI segmentation models. In this study, we aimed to develop 3D U-Nets for the tissue classification of anisotropic head CT, with larger slice thickness (~5 mm), and compared its CT brain tissue classification performance with 2D U-Nets. We trained both models using MR-derived segmentation labels and assessed the accuracy of the model by comparing CT segmentation results with MR segmentation results.

## Materials and Methods

### Datasets

We derived paired CT and MR datasets from the Gothenburg H70 Birth Cohort Studies. These multidisciplinary longitudinal epidemiological studies include six birth cohorts with baseline examinations at the age of 70 years to study the elderly population of Gothenburg in Sweden. For this study, we included same-day acquisitions of CT and MR images from 744 participants (52.6% female, mean age 70.44 ± 2.6 years) of the cohort born in 1944, collected from 2014 to 2016. The full study details are reported elsewhere (Rydberg Sterner et al., 2019). CT images were acquired on a 64-slice Philips Ingenuity CT system with a slice thickness of 0.9 mm, an acquisition matrix of 512 × 512, and a voxel size of 0.5 × 0.5 × 5.0 mm (Philips Medical Systems, Best, Netherlands). MRI scanning was conducted on a 3-Tesla Philips Achieva system (Philips Medical Systems) using a T1-weighted sequence with the following parameters: field of view: 256 × 256 × 160 voxels, voxel size: 1 × 1 × 1 mm, echo time: 3.2 ms, repetition time: 7.2 ms, and flip angle: 9° (Rydberg Sterner et al., 2019).

### Model Development and Training

#### Image Preprocessing

**Figure 1:**
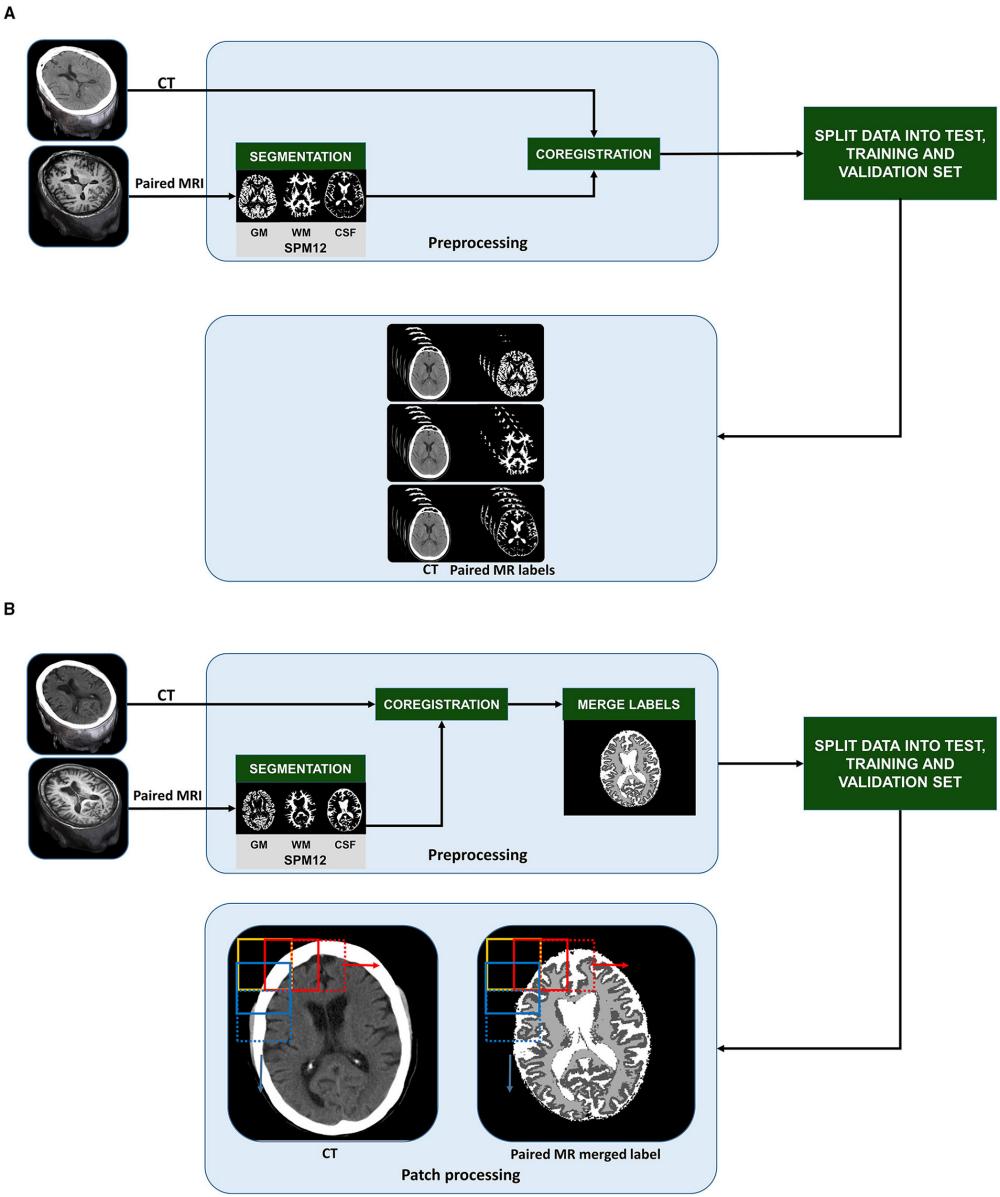
Pre-training stages of slice-wise processed 2D U-Nets **(A)** and patch-based 3D U-Nets **(B)**: CT and T1-weighted MR scans from 734 70-year-old individuals from the Gothenburg H70 Birth Cohort Studies were split into training (*n* = 400), validation (*n* = 100), and unseen test datasets (*n* = 234). In the pre-processing stage, MR images were segmented into gray matter (GM), white matter (WM) and cerebrospinal fluid (CSF) tissue classes. CT and MR labels were co-registered to each other. To create labels for multi task learning using 3D U-Nets, the MR labels were merged into a single label mask with 0 as background, 1 as GM, 2 as WM and 3 as CSF **(B)**. After splitting the datasets, the 3D CT and paired MR merged label volumes were split into smaller patches in a sliding fashion with a step of 64 in x and y direction (as indicated by the 2D representation of patch extraction shown by the yellow, red, and blue boxes). To create labels for single task learning using 2D U-Nets, after coregistration, the MR derived labels were organized slice wise **(A)**. Then, we created 3 group from the slices; input CT and paired MR-GM, input CT, and paired MR-GM, and finally, input CT and paired MR-CSF to train three separate 2D U-Net models for each tissue class.

We preprocessed all paired CT and MR images using SPM12 (http://www.fil.ion.ucl.ac.uk/spm), running on MATLAB 2020a (Figure 1), after first converting CT and MR images to NIfTI format and visually assessing the quality and integrity of all the scans. After that, each image was aligned to the anterior commissure- posterior commissure line.

We segmented MR images into GM, WM, and CSF labels using the unified segmentation algorithm in SPM12 (Ashburner and Friston, 2005). MR labels were used as training inputs to develop our models. To represent the CT images and MR labels in a common image matrix, MR images were coregistered to their paired CT images using SPM12 (Ashburner and Friston, 2007). One of the most decisive steps in our study is the coregistration between MRI and CT. A successful coregistration of MRI labels to CT scans enables MRI-derived labels to be used as training inputs. The difference in resolution affects the scaling and selection of starting points for the cost functions. SPM12 coregistration module optimizes the rigid transformation using a cost function, in our case, the normalized mutual information (Ashburner and Friston, 2007). Furthermore, the coregistration of each CT-MR pair was visually assessed, and 10 pairs were excluded based on faulty coregistration due to rotational or translational misregistration.

We developed 2D U-Nets to perform single-task learning and 3D U-Nets to perform multitask learning. To enable this, for 2D U-Net training, we paired each CT image with its corresponding MR-based GM, WM, and CSF labels (Figure 1A). For the 3D U-Net training, all the labels were merged into a single image and assigned the values 0 to the background, 1 to GM intensities, 2 to WM intensities, and 3 to CSF intensities (Figure 1B).

#### Data Preparation

We split the 734 processed datasets consisting of CT images and their paired, coregistered MR-label images into 500 training and 234 test datasets. We implemented a three-fold cross-validation in the training routine. The model hyperparameters were trained using the training datasets and fine-tuned with the validation dataset after each cycle or epoch and fold. The overall performance of the model was analyzed on the unseen test datasets. We used the same training and test dataset IDs for 2D and 3D U-Nets. Prior to the training routine, we thresholded all images within a 0–100 HU range, which is the recommended HU brain window. We resized the images to ensure that the length and width of the images remained 512, to accommodate the input size requirements of the U-Nets.

#### 3D U-Net Structure and Training

We performed all the patch-based processing steps (Figure 1A) in Python 3.7. The MR-merged labels were converted into categorical data to enable multitask learning. The 3D 512 × 512 × 32 sized CT images and categorical MR-merged labels were split into smaller 3D patches of size 128 × 128 × 32 by sliding through various layers in the image. For each paired image/label, we obtained 49 pairs of patches, thus obtaining 24,500 patches in the training routine.

We trained the 3D U-Nets using an Nvidia GeForce RTX 2080 Ti graphical processing unit (GPU), 11 GB of random access memory (Nvidia Corp., Santa Clara, CA, USA). The model architectures were developed and trained in Python 3.7, using TensorFlow 2.0 and Keras 2.3.1. The 3D U-Nets were developed to accept 3D patches of size 128 × 128 × 32.

**Figure 2:**
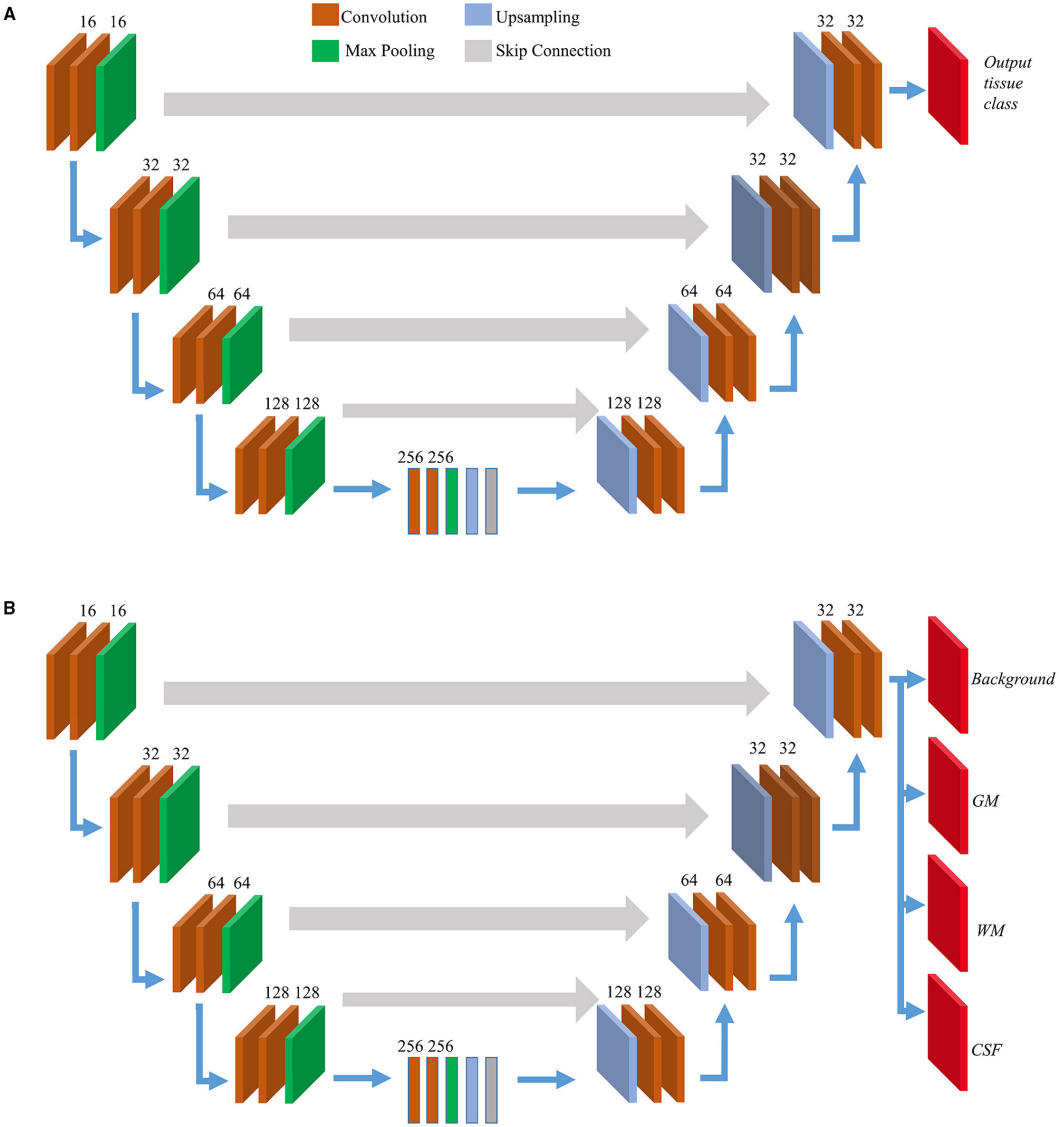
Model architectures. Overview of internal layers in 2D **(A)** and 3D U-Net **(B)** utilized to perform brain segmentation. A two or three-dimensional version of internal layers is used depending on slice-wise or patch-wise inputs. The output layer(s) depends on single or multi-task learning. For 2D U-Nets we used single-task learning, hence there was a single output layer. For 3D U-Nets, three tissue classes were trained at a time along with background hence there were four output layers.

The architecture of the 3D U-Net-based network is shown in Figure 2B. We resized all images to 512 × 512 × 32 to ensure that the spatial dimension of the output is the same as the input and to reduce the number of blank or empty patches. The U-Net architecture consisted of a contracting path or encoder path to capture context and a symmetric expanding path or decoder path that enables localization. In the first layer, the CT data were provided as input for training along with the corresponding ground truth, which in our case were the MR-merged labels. For 3D U-Nets, we programmed the internal functional layers using 3D functions. Each encoding block consists of two 3D convolutional layers with a kernel size 3 and a rectified linear unit (ReLU) activation function (Agarap, 2018), followed by 3D max-pooling and batch normalization layers. The number of feature maps increased in the subsequent layers to learn the structural features of various tissue classes. Dropout layers were added with a ratio of 0.2 to the last two encoding blocks.

We used symmetric decoding blocks with skip connections from corresponding encoding blocks and concatenated features to the deconvolution outputs. The final output layers were expanded to accommodate the multiclass nature of the ground truth labels. Categorical cross-entropy was used as the loss function, and a uniform loss was applied across all classification labels. We balanced the number of encoding/decoding blocks and patch size to accommodate the GPU memory constraints. Finally, four output maps with a 3D convolutional layer and a softmax activation (Dunne and Campbell, 1997) corresponding to the background, GM, WM, and CSF were generated. For this study, the learning rate was initialized to 0.0001. The adaptive moment estimation (Adam) optimizer (Kingma and Ba, 2014) was used, and all weights were initialized using a normal distribution with a mean of 0, an SD of 0.01, and biases as 0. After every epoch of the training routine, the predicted probability map derived from the validation is compared with its corresponding MR-merged label. Each voxel of the output segmentation maps corresponds to the probability of that voxel belonging to a particular tissue class. It calculates the error using the loss function, and it is backpropagated to the training parameters of the 3D U-Net layers. The learning saturated after 40 epochs and after this early stopping was implemented. The training time was approximately 25 h.

#### 2D U-Net Structure and Training

We developed and trained the 2D U-Net models using the procedures described in our previous study (Srikrishna et al., 2021). The architecture of the 2D U-Net-based network is shown in Figure 2A. We derived three models for GM, WM, and CSF tissue classification, respectively, using 2D U-Nets. In brief, we trained 2D U-Net-based deep learning models to differentiate between various tissue classes in the head CT. The segmentation patterns were studied from their paired MR labels. The 2D U-Net-based deep learning models were created and trained to accept CT scans and MR labels as input. The 2D U-Net follows the same structure as shown in Figure 2 with the internal functional layers programmed using 2D functions. The 2D U-Net was designed to perform the slice-wise processing of input data where each input image was processed as a stack of 2D slices with a size of 512 × 512 pixels. In total, 12,000 training slices and 3,000 validation slices were used to train 1,177,649 trainable hyperparameters. The inputs were fed into the model, and learning was executed using the Keras module. The batch size was 16. The callback features were used such as early stopping and automatic reduction of learning rate with respect to the rate of training. The model was trained for 50 epochs with 750 samples per epoch in approximately 540 min. We trained the 2D U-Nets also on an Nvidia GeForce RTX 2080 Ti GPU using TensorFlow 2.0 and Keras 2.3.1.

### Quantitative Performance Assessment and Comparison

For the quantitative assessment of the predictions from 3D U-Nets and 2D U-Nets trained on MR labels on the anisotropic CT data, we derived segmentation maps from both the deep learning models on the test datasets (*n* = 234) in Python 3.7, using TensorFlow 2.0 and Keras 2.3.1. The segmentations from test CTs were derived using deep learning models without the intervention of MRI and any preprocessing steps. Both models took <1 min to acquire prediction tissue class maps for one dataset; however, 2D U-Nets were 45 s faster than 3D.

For the comparative study, we used the same test datasets for both the 2D U-Nets and 3D U-Nets. We compared the predictions to their corresponding MR labels, which we employed as the standard or reference criterion. We adopted the approach suggested by the MRbrainS challenge (Mendrik et al., 2015), for the comparison of similarity between the predicted masks and standard criterion. We assessed the predictions using four measures, namely, continuous Dice coefficient (d_c_), Pearson's correlation of volumetric measures (*r*), Hausdorff distance (HD), and volumetric error (VE). The prediction maps from 2D U-Nets were obtained slice-wise. We stacked the slices to obtain 3D prediction maps for further comparison. We used the 3D version of all measures for the comparison of both 2D U-Net prediction maps and 3D U-Net predictions maps with their respective MR-derived labels.

To assess the spatial similarity between predicted probability maps and binary MR labels, we used the continuous Dice score, a variant of the Dice coefficient (Shamir et al., 2019). For distance similarity assessment, we used the average HD (AHD) and modified HD (MHD) (Dubuisson and Jain, 1994). We assessed the volumetric similarity using Pearson's correlation coefficient (Benesty et al., 2009) and VE. For this purpose, we binarized the prediction maps derived from both models. As the 3D U-Nets were trained using MR-merged labels, we used global thresholding with a threshold of 0.5 to binarize the predictions. For the prediction maps derived from 2D U-Nets, we used a data-driven approach for binarization. For image *I*, we derived segmentation maps *I*_*GM*_, *I*_*WM*_, and *I*_*CSF*_ using the 2D slice-wise predictions and stacked them to derive a 3D image. At each voxel *(x,y,z)*, we compared the intensities of *I*_*GM*_*(x,y,z), I*_*WM*_*(x,y,z)*, and *I*_*CSF*_*(x,y,z)*. We assigned the voxel *(x,y,z)* to the tissue class with maximum intensity at that voxel. This method ensures that there are no overlapping pixels among various tissue classes. We measured VE by deriving the absolute volumetric difference divided by the sum of the compared volumes.

## Results

**Figure 3:**
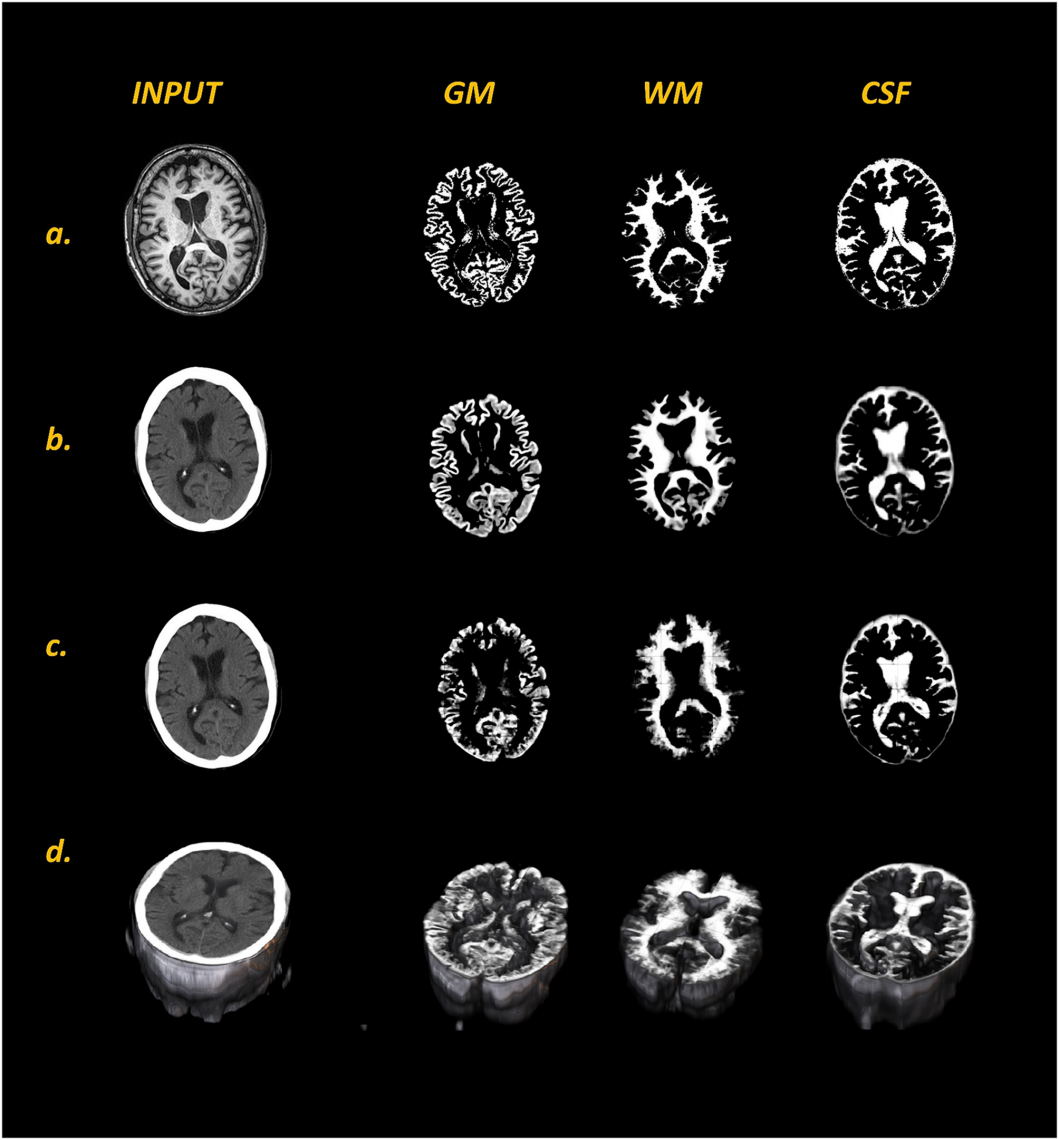
Comparison of MR labels **(a)** with respective input CT images and predicted tissue class maps (GM, WM, and CSF) generated with 2D U-Net **(b)** and 3D U-Net **(c)** models from a representative dataset. Panel **(d)** shows the 3D visualization of input CT and 3D U-Net predicted tissue class maps. In comparison to 2D U-Nets, 3D U-Nets was not able to resolve the finer details of all three tissue classes, especially WM.

The representative predicted segmentations of different tissue classes derived from test CTs by 2D U-Nets and 3D U-Nets are shown in Figure 3. Table 1 presents the resulting metrics obtained from our comparative analysis. We compared the predictions from 2D U-Nets and 3D U-Nets separately with MR-derived labels.

**Table 1:** Quantitative performance metrics in test datasets (*n* = 234).

**Assessment metrics**	**Model**	**GM**	**WM**	**CSF**
d_c_	2D U-Nets 3D U-Nets	0.80 ± 0.03 0.59 ± 0.02	0.83 ± 0.02 0.65 ± 0.03	0.76 ± 0.06 0.55 ± 0.05
r	2D U-Nets 3D U-Nets	0.92 0.85	0.94 0.73	0.9 0.73
VE	2D U-Nets 3D U-Nets	0.07 ± 0.03 0.09 ± 0.08	0.03 ± 0.03 0.04 ± 0.12	0.07 ± 0.06 0.11 ± 0.13
AHD (mm)	2D U-Nets 3D U-Nets	4.60 ± 1.6 7.03 ± 2.6	4.10 ± 1.8 13.49 ± 6.1	4.43 ± 1.5 8.34 ± 7.2
MHD (mm)	2D U-Nets 3D U-Nets	1.67 ± 1 2.59 ± 1.4	1.19 ± 0.8 4.29 ± 2.9	1.42 ± 0.6 4.65 ± 3.9
V_CT_ (liters)	2D U-Nets 3D U-Nets	0.55 ± 0.07 0.81 ± 0.11	0.52 ± 0.08 0.64 ± 0.12	0.27 ± 0.05 0.44 ± 0.09

*Continuous Dice score (d_c_) expresses the extent of spatial overlap between CT predictions from 2D U-Nets/3D U-Nets and MR labels, Pearson's correlation coefficient (r) measures the linear relationship between volumetric measures, AHD and MHD are distance measures, and volumetric error (VE) expresses the absolute volumetric difference between CT segmentation maps and MR labels. The volumes of CT predictions are given by V_CT_, respectively*.

**Figure 4:**
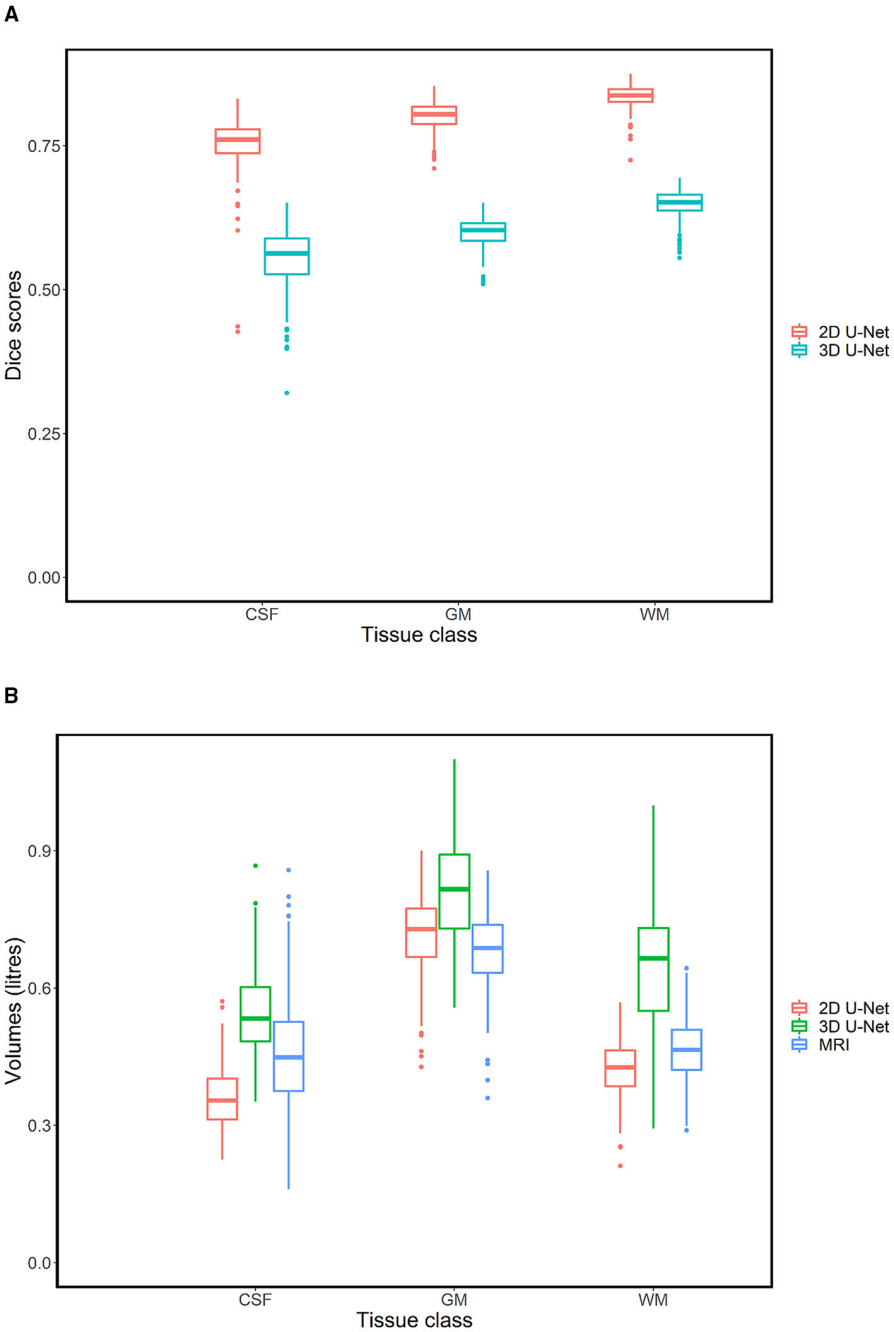
Box plots showing differences in dice scores **(A)** and volumes **(B)**. CT-derived segmentations produced by 2D U-Nets had much better spatial overlap with its paired MR labels in comparison to CT-derived segmentations from 3D U-Nets. With respect to the MR-derived volumes, 3D U-Nets over estimated volumes in all tissue classes in comparison to 2D U-Nets.

In comparison with 3D U-Nets, 2D U-Nets performed better in the segmentation of all three tissue classes. The 2D U-Nets yielded d_c_ of 0.8, 0.83, and 0.76 in GM, WM, and CSF, respectively. The boundary measures expressed by AHD, MHD (in mm) were found to be (4.6, 1.67), (4.1, 1.19), and (4.43, 1.42) for GM, WM, and CSF, respectively. Pearson's coefficient for volumetric measures was observed to be 0.92, 0.94, and 0.9 for GM, WM, and CSF, respectively, with a VE of 0.07, 0.03, and 0.07. In the case of 3D U-Nets, we yielded d_c_ of 0.59, 0.65, and 0.55 in GM, WM, and CSF, respectively. The boundary measures expressed by AHD, MHD (in mm) were found to be (7.03, 2.59), (8.49, 4.29), and (8.34, 4.65) for GM, WM, and CSF, respectively. Pearson's coefficient for volumetric measures was observed to be 0.85, 0.73, and 0.73 for GM, WM, and CSF, respectively, with a VE of 0.09, 0.04, and 0.11. Figure 4 shows the comparative performance of Dice scores and volumes between 2D U-Nets and 3D U-Nets. Volume predictions from 3D U-Nets were overestimated in comparison with predictions from 2D U-Nets in all tissue classes.

## Discussion

Our previous study showed that 2D deep learning-based algorithms can be used to quantify brain tissue classes using only CT images (Srikrishna et al., 2021). In this study, we explored the possibility of using 3D-based deep learning algorithms for CT brain tissue classification and compared the performance of 2D- and 3D-based deep learning networks to perform brain tissue classification in CT scans. Our CT images are anisotropic, with low resolution (5 mm) along the z-axis. This study shows that 2D segmentation networks are appropriate to deal with datasets of this nature. This is the only study that compares 2D and 3D segmentation networks for 1D CT data showing that the type of model architecture and learning approach is dependent on the nature of the input CT datasets.

To process our CT datasets with 3D networks, either we would have to downsample the whole CT image to a size to suit the available memory or we would process the image in smaller patches. In modalities such as MRI, where the contrast resolution is high, resizing would not affect the images. However, in the case of CT, where the contrast resolution is lower, we observed that resizing further reduced the contrast resolution of the CT images. Hence, we concluded that downsampling CT images sacrifice potentially important information in the model training. Therefore, we opted for sliding patch-wise 3D processing. For our patch-based 3D U-Nets to train effectively, we attempted various patch sizes and U-Net depth. We opted for the patch size of 128 × 128 × 32, given that a lower patch size limits the receptive field the deep-learning network can notice, whereas a higher patch size has more memory requirements.

We compared the performance of 2D and 3D CT-based segmentation networks with respect to MR-derived labels using measures of distance, overlap, and volume. Overall, 2D U-Nets performed better than 3D U-Nets in all measures and all tissue classes. In terms of volume, the 2D and 3D U-Nets showed comparable performance (Table 1). However, 3D U-Net generally overestimated volume predictions for all tissue classes in comparison with 2D U-Nets. In terms of spatial overlap and distance measures, the single-task-based, slice-wise processed 2D U-Nets performed much better than multitask- and patch-wise processed 3D U-Nets. We attributed this to the nature of the dataset. The main purpose of using 3D U-Nets for 3D data is to capture contextual information in all planes and directions. However, for our datasets, where the thickness along the z-axis is large (5 mm), only limited contextual information can be derived from the z-axis. For such data, maximum information is present in one plane, in our case, the axial plane. Therefore, processing the data slice-wise and segmenting using 2D segmentation networks are more useful than processing patch-wise with 3D segmentation networks. In future, we aim to test this further by slicing the volume along 1D coronal or sagittal CT scans and by comparing the resulting U-Nets.

Various automated approaches and evaluation methods for CT segmentation have been described previously. Gupta et al. (2010) used domain knowledge to improve segmentation by adaptive thresholding, and Kemmling et al. (2012) created probabilistic atlas in standard MNI152 space from MR images. These atlases were transformed into CT image space to extract tissue classes. Cauley et al. (2018) explored the possibility of direct segmentation of CT using an MR segmentation algorithm employed in FSL software. CT segmentations derived from these algorithms lacked sharpness, and some of these results were not validated against manual or MR segmentations or using standard segmentation metrics for comparison. Our 2D U-Net model outperforms the study by Manniesing et al. (2017), which uses four-dimensional (4D) CT to create a weighted temporal average CT image, which is further subjected to CSF and vessel segmentation followed by support vector-based feature extraction and voxel classification for GM and WM segmentation, in terms of Dice coefficients and HDs. Our 3D U-Nets outperforms this study in terms of HDs, which were 14.85 and 12.65 mm for GM and WM, respectively, but underperformed in terms of Dice coefficients.

One of the unique features of our study is the nature of the datasets and the usage of MR-derived labels from MR-based automated segmentation tools as the standard criterion. Even though manual annotations are considered the “gold standard” for CT tissue classification, manual annotations are labor-intensive, rater-dependent, time-consuming, and challenging to reproduce. In the case of our study, we had access to a unique cohort with a large number of CT and MRI datasets collected close to each other in time. The MR-derived labels are apt for the localization and classification of brain tissue. They are easy to extract using reliable and readily available automated software such as FreeSurfer, SPM, and FSL even for large cohorts such as the H70 Birth Cohort.

In terms of neurodegenerative disease assessment, CT and structural MRI are both commonly used for visual assessments. However, even though both of these modalities are commonly used, we cannot ensure that deep learning networks, which have performed successfully in MR-based segmentation, can show similar performance in CT-based segmentation. For instance, in a few studies, patch-based 3D U-Nets have been successfully used for MR-based segmentation (Ballestar and Vilaplana, 2020; Qamar et al., 2020); however, in the case of anisotropic CT-based brain segmentation, 2D U-Nets performed better.

In addition to all these strengths, our study also has some limitations. The choice of using MR-based labels as training inputs has its advantages and disadvantages. In our previous study (Srikrishna et al., 2021), we attempted to understand the difference in the segmentations derived from the two modalities. Our previous studies show that performing brain segmentation with CT instead of MRI incurs a loss of accuracy similar to or less than that of performing brain segmentation on an MR image that has been degraded through the application of Gaussian filtering with an SD of 1.5. The effect of this difference on the development of segmentation models and comparison of MR labels and manual labels as training inputs for these segmentation models needs to be further explored. However, it is a challenging task to derive manual labels for tissue classes such as GM, WM, and CSF in large cohorts. Currently, we only compared U-Net-based segmentation networks. In future studies, we aim to train and compare various state-of-the-art 2D segmentation networks for CT datasets. We also plan to compare various 2D segmentation network architectures, loss functions, and learning methods, as well as find the optimum features for brain tissue class segmentation in CT. We also plan to increase our training data and validate our study in several cohorts since the training on one cohort might not necessarily give the same results in another (Mårtensson et al., 2020). We plan to explore the possibility of using 2.5D segmentation networks for 1D CT brain tissue classification and study the effect of a skull in the segmentation performance in CT scans. We plan to conduct the clinical validation of CT-derived measures for neurodegenerative disease assessment and compare the diagnostic accuracy of CT-based volumes derived using various segmentation algorithms.

## Data Availability Statement

The datasets presented in this article are not readily available because data from the H70 cohort cannot be openly shared according to the existing ethical and data sharing approvals; however, relevant data can and will be shared with research groups following the submission of a research proposal to and subsequent approval by the study leadership. Requests to access the datasets should be directed to Michael Schöll, michael.scholl@neuro.gu.se.

## Ethics Statement

The studies involving human participants were reviewed and approved by the H70 study was approved by the Regional Ethical Review Board in Gothenburg (Approval Numbers: 869-13, T076-14, T166-14, 976-13, 127-14, T936-15, 006-14, T703-14, 006-14, T201-17, T915-14, 959-15, and T139-15) and by the Radiation Protection Committee (Approval Number: 13-64). The patients/participants provided their written informed consent to participate in this study.

## Author Contributions

MS contributed to conceptualization, methodology, software, formal analysis, data curation, and writing-original draft preparation. RH and JP contributed to supervision, methodology, and writing-reviewing and editing. GV contributed to methodology and writing-reviewing and editing. AZ and SK contributed to data generation and reviewing and editing. EW contributed to conceptualization and reviewing and editing. IS contributed to funding acquisition, data generation, and reviewing and editing. MS contributed to funding acquisition, conceptualization, supervision, project administration, and writing-reviewing and editing. All authors contributed to the article and approved the submitted version.

## Funding

The Gothenburg H70 Birth Cohort 1944 study was financed by grants from the Swedish state under the agreement between the Swedish government and the county councils, the ALF-agreement (ALF 716681), the Swedish Research Council (2012-5041, 2015-02830, 2019-01096, 2013-8717, and 2017-00639), the Swedish Research Council for Health, Working Life, and Welfare (2013-1202, 201800471, AGECAP 2013-2300, and 2013-2496), and the Konung Gustaf V:s och Drottning Victorias Frimurarestiftelse, Hjärnfonden, Alzheimerfonden, Eivind och Elsa K:son Sylvans Stiftelse. This study was supported by the Knut and Alice Wallenberg Foundation (Wallenberg Centre for Molecular and Translational Medicine; KAW 2014.0363), the Swedish Research Council (#201702869), the Swedish state under the agreement between the Swedish government and the County Councils, the ALF-agreement (#ALFGBG-813971), and the Swedish Alzheimer's Foundation (#AF740191). This work used computing resources provided by the Swedish National Infrastructure for Computing (SNIC) at Chalmers Center for Computational Science and Engineering (C3SE), partially funded by the Swedish Research Council through grant agreement no. 2018-05973.

## Conflict of Interest

The authors declare that the research was conducted in the absence of any commercial or financial relationships that could be construed as a potential conflict of interest.

## Publisher's Note

All claims expressed in this article are solely those of the authors and do not necessarily represent those of their affiliated organizations, or those of the publisher, the editors and the reviewers. Any product that may be evaluated in this article, or claim that may be made by its manufacturer, is not guaranteed or endorsed by the publisher.
